# DUCU: a conceptual framework for AI-driven conversion of smile design to functional wax-up

**DOI:** 10.3389/froh.2025.1690479

**Published:** 2025-11-19

**Authors:** Sergiu Drafta, Andrei Macris, Alexandru E. Petre

**Affiliations:** Prosthetic Department, Dentistry Faculty, Carol Davila University of Medicine and Pharmacy, Bucharest, Romania

**Keywords:** digital smile design, artificial intelligence, CAD/CAM prosthetics, aesthetic smile simulation, digital wax-up

## Abstract

With the rapid evolution of esthetic digital dentistry, patient-centered tools were developed, such as digital smile design, to help improve patient communication and emotional participation. However, a major difference remains between these purely aesthetic simulations and the functional accuracy necessary with computer-aided design/computer-aided manufacturing restorative workflows. In this paper, we present the conceptual basis for a new, intelligent, computer-aided design/computer-aided manufacturing (CAD/CAM) wax-up design theory called Dental Unified CAD Utility (DUCU). This platform includes advanced artificial intelligence algorithms for tooth morphology, margin detection, intaglio surface generation, and occlusal correspondence, to combine emotional aesthetics with clinical function. Through facilitation of interdisciplinary teamwork and automation of restorative design workflows, Dental Unified CAD Utility sets out to drastically decrease laboratory time, reduce human errors, and boost treatment predictability. We describe implications and barriers to clinical implementation and future research directions necessary for the development and validation of the DUCU as a transformative tool in digital prosthodontics.

## 1 Introduction

The digital transformation of dentistry has changed the way we work clinically, such as aesthetic planning and prosthodontics. One of the most impacting is the introduction of the digital smile design (DSD) (1) protocol that provides clinicians with a comprehensive method to treatment plan and visualize the smile and dent esthetics using both two-dimensional (2D) and three-dimensional (3D) facial analysis tools. These systems, including platforms such as SmileCloud, have greatly facilitated patient communication, interdisciplinary planning, and case acceptance by integrating dental restorations based on the patients' facial aesthetics and natural tooth libraries (2).

Novel developments in artificial intelligence (AI), especially by means of deep-learning (3) algorithms and neural network structures, have also greatly facilitated digital dentistry. These AIs can automatically do image identification, precise anatomic modeling, intelligent margin detection, and dynamic occlusion mapping, which greatly improve the diagnostic discrimination and efficiency (4–10). Even with such developments, a significant disconnect still persists; the gap between visual smile design and the restorative computer-aided design/computer-aided manufacturing (CAD/CAM) phase is not customarily bridged. Smile designs, although resonant with emotion and facial anatomic landmarks, are frequently rendered as skin-deep appearances—without functional anatomy, occlusion relationships, and inter-arch morphology integration—for fabrication in the living environment. As a result, dental technicians and clinicians need to manually reinterpret smile mock-ups into functional wax-ups, which is a time-consuming, skill-dependent, and inevitably subjective operation (9, 10).

**Figure 1:**
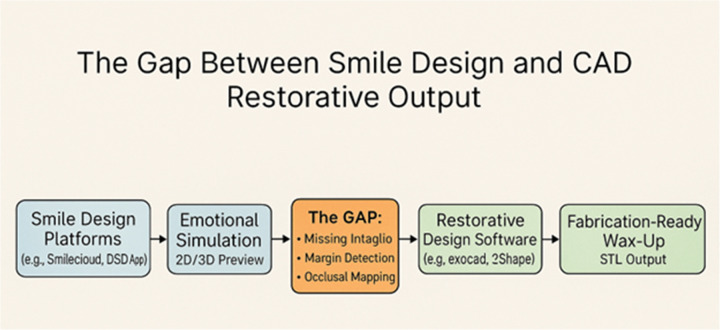
Discrepancy between smile design and CAD restorative output.

This void constrains the scope, speed, and reliability of today's aesthetic dentistry, especially for cases requiring a measure of patient engagement, while also mandating accurate restorative execution. At present, there is no method available for automated conversion of a DSD-style smile simulation into an anatomically correct, growth mark-driven, margin-rich, printable restoration (Figure 1) (2, 11, 12).

In the present work, we introduce Dental Unified CAD Utility (DUCU)—an AI-driven, next-generation framework that aims to narrow the gap between aesthetics and functionality. DUCU is conceptually envisaged as an intelligent, cloud-connected system that can convert simple, superficial smile designs to full 3D restorations, automatically design functional intaglio surfaces, identify preparation margins from intraoral images, predict static and dynamic occlusion (13), and produce CAD/CAM-ready standard tessellation language (STL) files with the details required for either 3D printing or milling. Integrating aesthetic simulation and prosthetic precision, DUCU offers a new digital smile design workflow for patients and dental team collaboration (14–16).

## 2 Dental Unified CAD Utility (DUCU)

### 2.1 Problem statement

DSD software has allowed clinicians to visualize a pleasant treatment outcome and help to increase patient cooperation by creating a lucrative case presentation. Such tools, although endowed with excellent capacities for representation of visual narrative and facial-based planning, are yet separated from the minute technical challenges required for restorative design and production. Aesthetic smile simulations are usually skin-deep models not possessing the essential anatomical, functional, and material-based features necessary to be directly integrated in CAD/CAM restorative workflows (17).

A number of serious constraints result in the clinic owing to this disconnect. First, many of today's smile simulations are found without a proper definition of the intaglio surfaces that are necessary for an accurate fit on tooth preparations and for an unnoticeable link with the gingiva. Such a deficiency may compromise the fit of the restoration and pose the risk of clinical issues, such as marginal leakage or gingival irritation.

Second, these simulations often lack accurate clinical margin definition, requiring dental technicians to manually adjust or reinterpret designs to accommodate specific tooth preparations. Such a manual operation not only introduced subjective and objective factors but also extended laboratory time, increased laboratory costs, and added damage or mistakes for restoration.

Lastly, the majority of aesthetic simulations are made without taking occlusal dynamics into account and fail to address salient issues, such as inter-arch relation, vertical dimension, and functional paths of movement. This negligence may result in functional discrepancies with the final restorations and the necessity of further modifications at the chairside, compromising patient comfort and long-term restoration survival.

Finally, in their workflow, dental laboratories must often deal with a non-negligible workflow inefficiency of rebuilding the proposed smile workflows completely from the beginning within a generalized CAD/CAM software and lose a connection between clinical and technical vision.

In conclusion, there is no current system that automates the conversion of a facial-driven smile simulation to a finished, printable, millable, and clinically useful prosthetic restoration. This lack of integration of the aesthetic design process and functional prosthodontic need is a significant obstacle to the adoption of fully digital workflows in aesthetic restorative dentistry.

### 2.2 DUCU theoretical and conceptual framework

**Figure 2:**
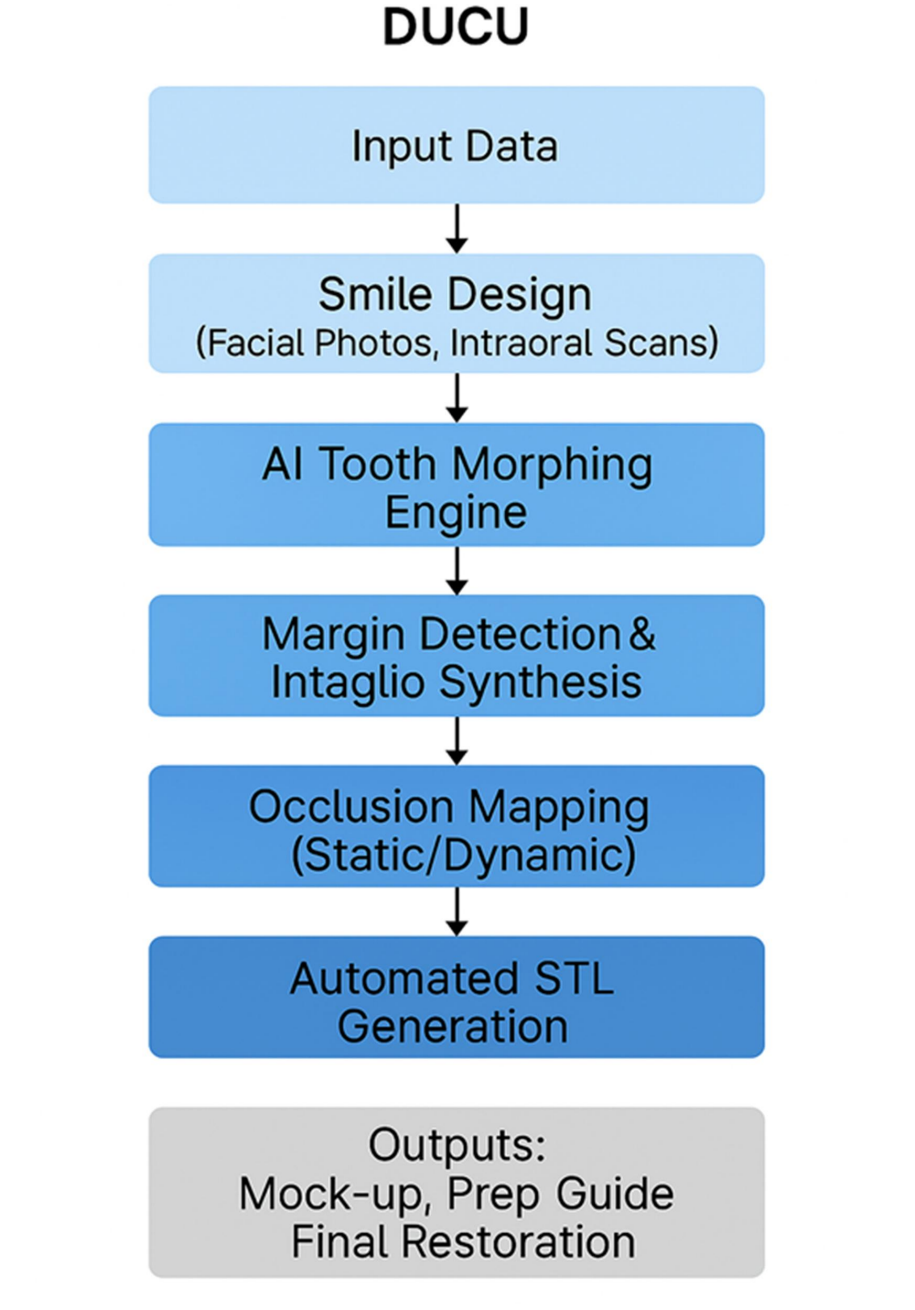
DUCU overall workflow.

To bridge this gap between emotionally driven smile design and CAD/CAM prosthetics in better performing, we propose DUCU: a conceptual AI-based framework, aiming to automatically convert smile simulations driven by the face to an anatomic, precise, functionally validated, digital wax-up, ready for fabrication. DUCU is considered a cloud-based AI-powered software framework that interconnects dentists, dental technicians, and patients under one system (Figure 2).

**Figure 3:**
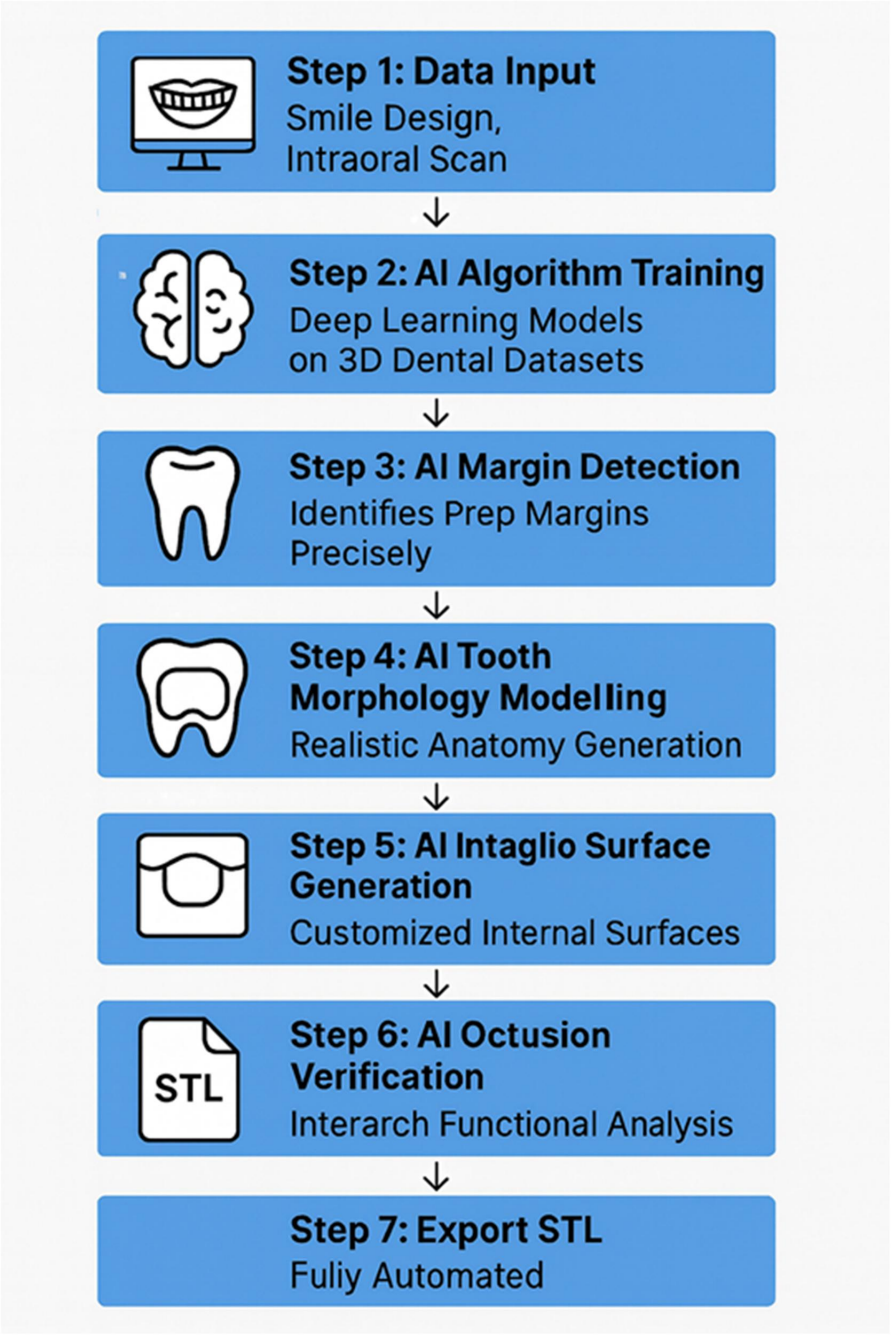
Detailed AI-driven transformation process.

The main part of DUCU offers an AI tooth morphing engine to mimic natural anatomy from smile designs, inlay and intaglio synthesis to fit upscaled margin lines with checked clinical prepared data, occlusion (13) mapping based on antagonist scans or jaw recordings, and final restoration preparation guide-rich export-ready STL generation (mock-up, prep guide, final restorations). The system would also provide a shared cloud interface to integrate treatment planning and delivery among stakeholders (Figure 3) (18, 19).

Current solutions, such as SmileCloud (SmileCloud SRL, Timișoara, Romania), DSD App (DSD Planning Center S.L., Madrid, Spain), and exocad (2) (exocad GmbH, Darmstadt, Germany), work under the tacit premise that visual simulation tools, if approved by the patient, may be translated into a restorative workflow, using manual technician expertise. Although these have significantly improved communication of emotions and case acceptance, their ability to directly produce a functional, production-compatible product is restricted. DUCU contests this simplicity regarding aesthetic design as the tendency to transform the design to be necessarily integrated with anatomical accuracy, margin accuracy, and occlusal logic through an AI-based system. In doing so, it reframes the clinical workflow as something that needs to be “testable,” “predictable,” and “automatable” within the existing technological infrastructure of digital dentistry.

Currently, DUCU exists as a conceptual and architectural prototype developed in a Python environment. It integrates deep-learning frameworks (PyTorch, TensorFlow) and a 3D modeling library (Open3D) to simulate proof-of-concept modules for AI-driven margin detection, morphology synthesis, and virtual wax-up generation. Although DUCU is not yet a standalone program with autonomous restorative design capabilities, it establishes the algorithmic foundation for future AI-assisted dental CAD workflows.

**Figure 4:**
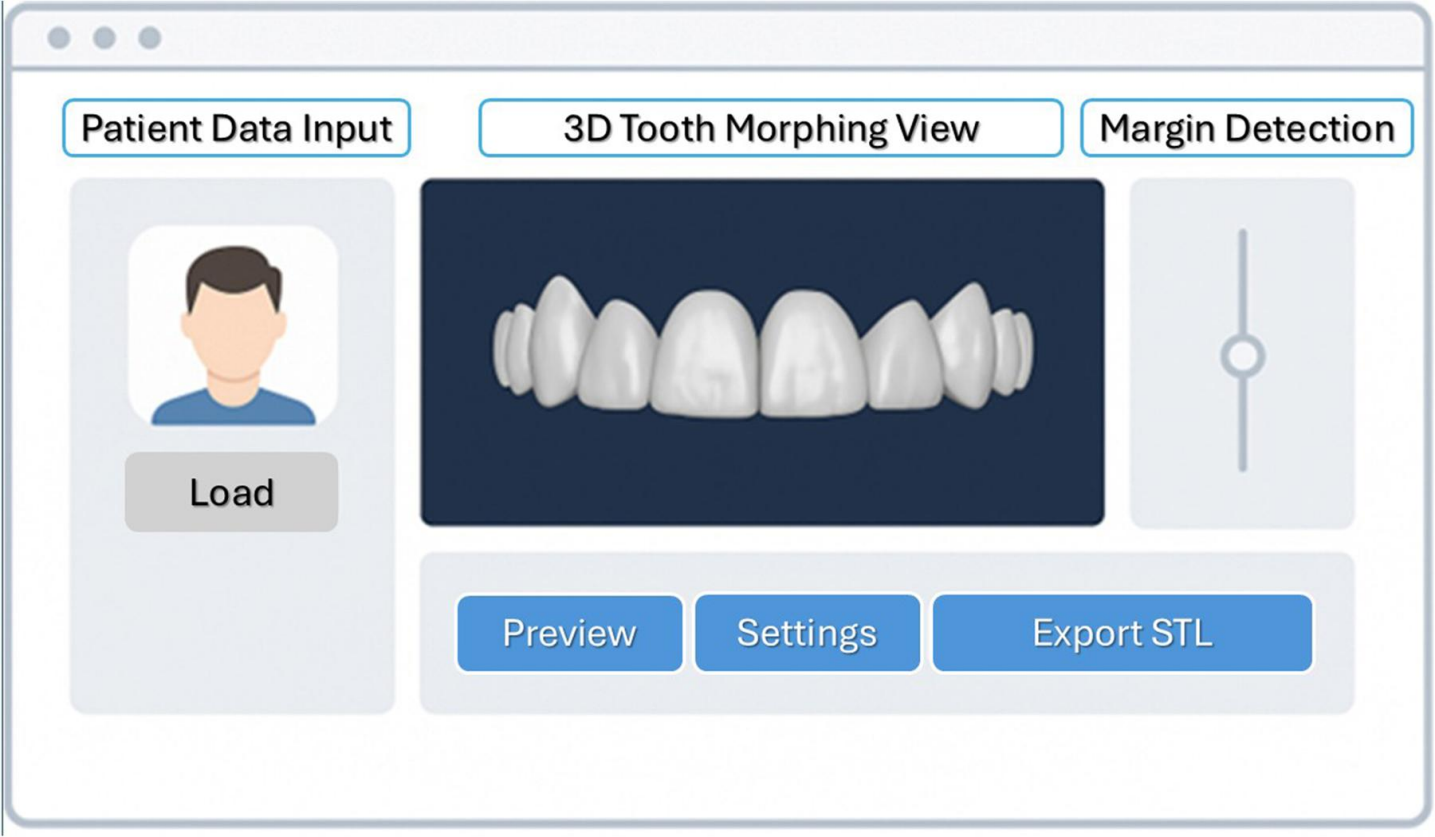
Early prototype mock-up of the DUCU framework interface showing the main AI-assisted modules.

Figure 4 illustrates the early prototype mock-up, highlighting the main interface components: Patient Data Input, 3D Tooth Morphing View, AI Margin Detection Panel, and STL Export Controls. This schematic demonstrates how DUCU conceptually integrates data acquisition, artificial intelligence, and output generation within a unified digital framework (Figure 4). In this context, the term mock-up refers to the prototype interface of the DUCU software and should not be confused with the clinical wax-up, which designates the functional restorative model generated by the system.

There are several key pillars to a successful DUCU. First, we need to build a team that is capable in this kind of specialized interdisciplinary task that includes AI/ML (artificial intelligence/machine learning) engineers with 3D deep learning (2) and segmentation and dental-specific AI dataset expertise; full-stack developers in web and desktop (React, Python, Electron, Node.js) and 3D rendering; CAD/CAM platform engineers with knowledge of dental standard tessellation file/object file (STL/OBJ) mesh processing, margin detection, and intaglio generation; development operations (DevOps) and cloud engineers responsible for secure Amazon Web Services (AWS)/Google Cloud Platform (AWS/GCP) deployment and maintaining compliance standards, such as Health Insurance Portability and Accountability Act (HIPAA) or General Data Protection Regulation (GDPR); user interface/user experience (UI/UX) designers focused on designing intuitive workflows for dental professionals; dental consultants, such as prosthodontists or laboratory technicians to drive clinical protocols and output quality; and project/product management to ensure technology and clinical needs align.

Second, it is important to have a strong technology stack. This includes AI frameworks such as PyTorch (PyTorch Foundation, Linux Foundation, San Francisco, USA) and TensorFlow (Google LLC, Alphabet Inc., Mountain View, USA), 3D libraries such as Open3D (Open 3D Foundation, Linux Foundation, San Francisco, USA), Trimesh (TriMesh library, Michael Dawson-Haggerty), MeshLab (Visual Computing Lab of Istituto di Scienza e Tecnologia dell'Informazione—Consiglio Nazionale delle Ricerche, University of Pisa, Pisa, Italy), and Point Cloud Library (Willow Garage, under Berkeley Software Distribution license) for tooth morphology modeling and margin detection. Data files, CAD/CAM integration, and data file processing include integration with diverse file types based on an STL, OBJ, or a polygon file format (PLY) and use of available dental software development kits (SDKs) such as from exocad (exocad GmbH, Darmstadt, Germany), 3Shape (3Shape A/S, Copenhagen, Denmark), and Medit (Medit Co, Seoul, South Korea). Furthermore, occlusion-in-simulation technologies using static and dynamic virtual articulators are expected to improve clinically generated functionality. The platform architecture would feature front tools such as React (Meta Platforms Inc., Menlo Park, CA, USA), Three.js (Ricardo Cabello, Barcelona, Spain), WebGL (Khronos Group Inc., Beaverton, USA), or Unity (Unity Software Inc., San Francisco, USA), frontend and backend performed in Python (Python Software Foundation, Wilmington, USA) through web services (Flask or FastAPI), with management of databases through PostgreSQL (a free and open-source relational database management system) or Firebase (Google LLC, Alphabet Inc., Mountain View, USA), and the deployment of cloud services using AWS (Amazon Inc., Seattle, USA), such as EC2, S3, and Cognito/Auth0.

Third, a full data and infrastructure planning is required. Standardization of critical datasets (such as intraoral scans sourced from clinical collaborations or public repositories, examples of smile design landing uses from services such as SmileCloud, and fitted and approved final restorations with marked margins) must be performed. Some of the basic infrastructure is to have a graphic processing unit (GPU)-based mashed for training the AI models, a strong versioning control system (GitHub, GitLab, etc.), and good project management tools (Notion, Jira, Trello, etc.) and to be compliant with data protection regulations (HIPAA, GDPR, etc.).

Finally, an effective launch and growth strategy is necessary. Phase 1, Minimum Viable Product (MVP) (0–6 months) would focus on developing basic AI-driven smile simulation capabilities with STL file export functionality. This site remains funded at null cost, and beta release activities (6–9 months) will include phase 2 of learning the occlusion simulation, compatibility with lab software such as exocad (2), and setting up cloud storage of patient cases, as well as ongoing working with early adopters in the dental community. The commercial stage (9–12 months) would include securing regulatory clearances, including CE/FDA acts for class I software, establishing a sustainable subscription-based business model, partnering with digital scanner manufacturers, and providing advanced features such as augmented reality (AR) viewers to improve patient interactions (20).

DUCU is envisioned as a collaborative, cloud-based framework integrating diverse professional perspectives—prosthodontic, orthodontic, periodontal, technical, and computational. Within this model, the prosthodontist interprets and encodes the patient's emotional and aesthetic expectations as measurable design parameters, linking subjective perception with digital planning. The orthodontist and periodontist contribute diagnostic boundaries that preserve biomechanical and biological integrity, while the dental technician ensures material feasibility and esthetic consistency. The engineer or AI specialist continuously refines algorithms based on aggregated feedback from all disciplines.

**Figure 5:**
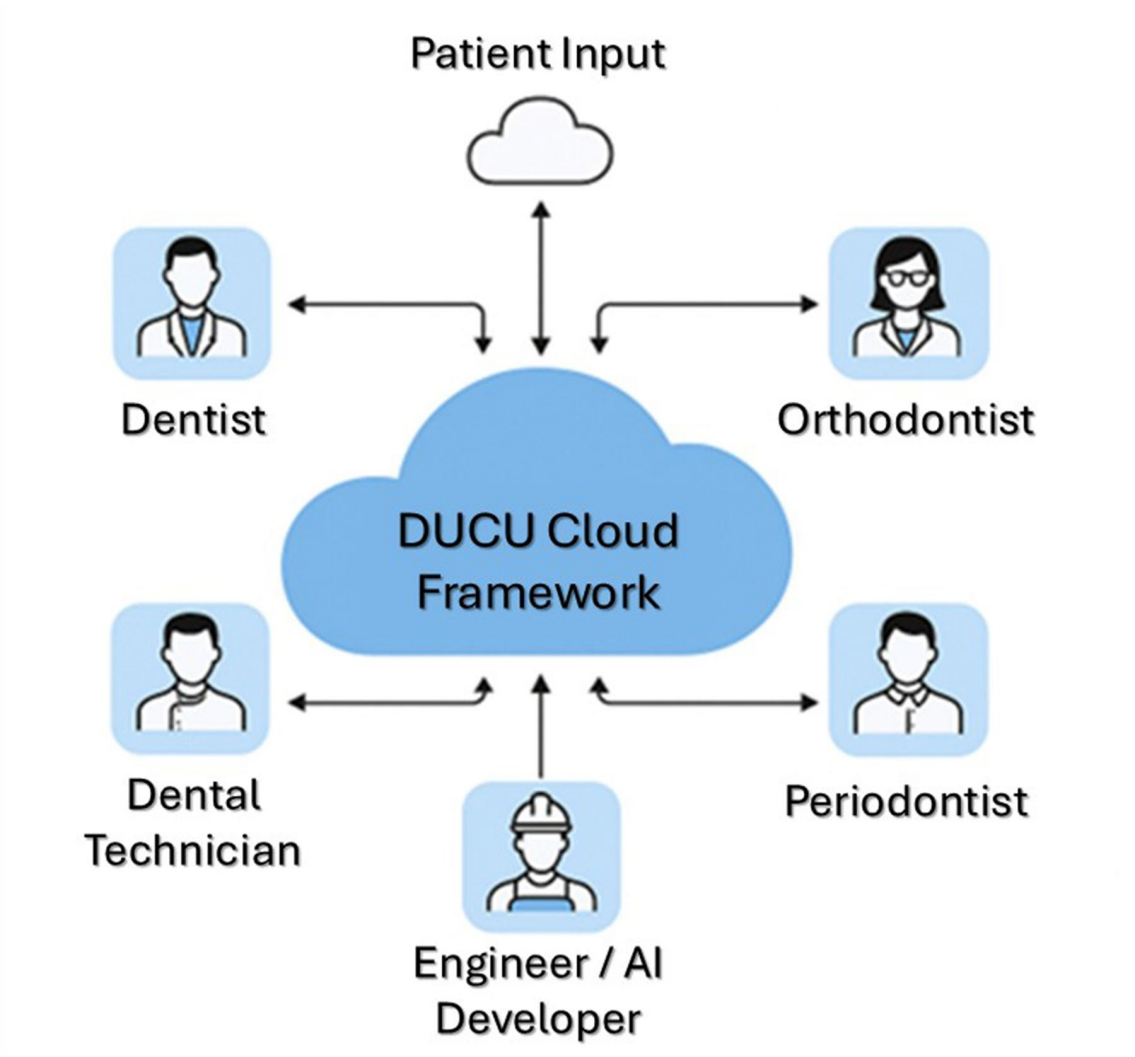
Conceptual diagram showing how DUCU connects multiple specialists through a unified cloud platform for synchronized data exchange.

This interdisciplinary model conceptually merges emotional aesthetics, clinical function, and computational intelligence into a unified virtual workflow, where all contributors interact with the same dataset in real time through the DUCU cloud infrastructure. Figure 5 conceptually illustrates how DUCU integrates emotional aesthetics and clinical data from multiple specialists into a shared, cloud-based workflow, promoting balanced decision-making between aesthetic intention and functional predictability.

Possible further improvements may include, for example, automated preparation guide generations, automatic diagnostic wax-ups, digital twin patient records systems, and multiuser collaborative environments for full mouth dental care.

This efficient process integrates the historically disjointed smile-to-restoration flow into one seamless, smart system. From design to STLs in a click, DUCU is the next evolution in restorative digital dentistry.

### 2.3 Theoretical use-case scenario

Consider a 34-year-old woman who presents with chief concerns about worn incisal edges, anterior asymmetry, and low smile confidence as a request for a smile makeover consultation. At present, the traditional process would be divided into two manual steps: we take high-resolution facial photos and intraoral scans, and then we create a manual smile simulation and show the patient. Once approved, the case design would have to be manually reconstructed using CAD, with repositioning margins, occlusion (13), and intaglio surfaces with all the hazards of human interpretation.

**Figure 6:**
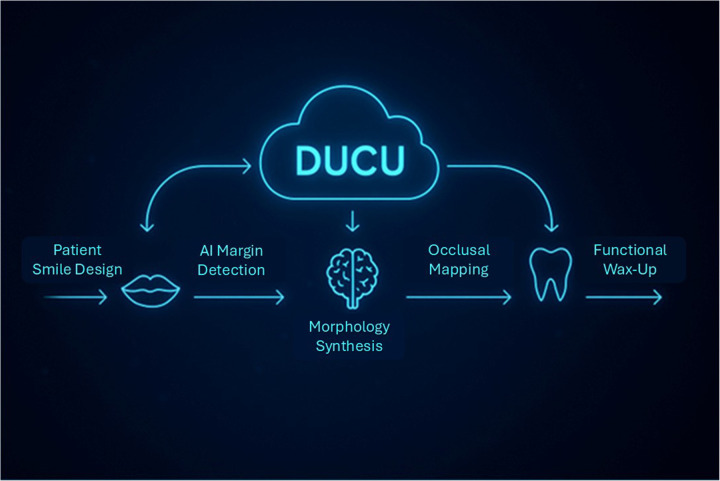
The conceptual flow of the DUCU framework, showing the theoretical transition from patient smile design to AI-informed morphology synthesis and functional wax-up representation.

This is where DUCU comes into play. Following the patient's aesthetic smile simulation approval, complete margins are notated in DUCU, and precise intaglio surfaces are generated by the software for a close morphing of the aesthetic simulation into a full anatomically accurate wax-up (13) for functional occlusal verification with the antagonist scan (Figure 6). Printable STL files for motivational mock-ups, preparation guides, and final restorations are created effortlessly without reinterpretation by the user. This translates into a substantial time saving in the laboratory, improved accuracy, and fully developed rehabilitations that will function and look as close as possible to the patient's accepted simulation.

To better illustrate the proposed clinical workflow, conceptual simulations were developed to visualize how DUCU could operate once implemented. These models are not derived from an existing software output but are illustrative representations built from anonymized design data, aimed at depicting the theoretical stages of AI-assisted restorative planning.

**Figure 7:**
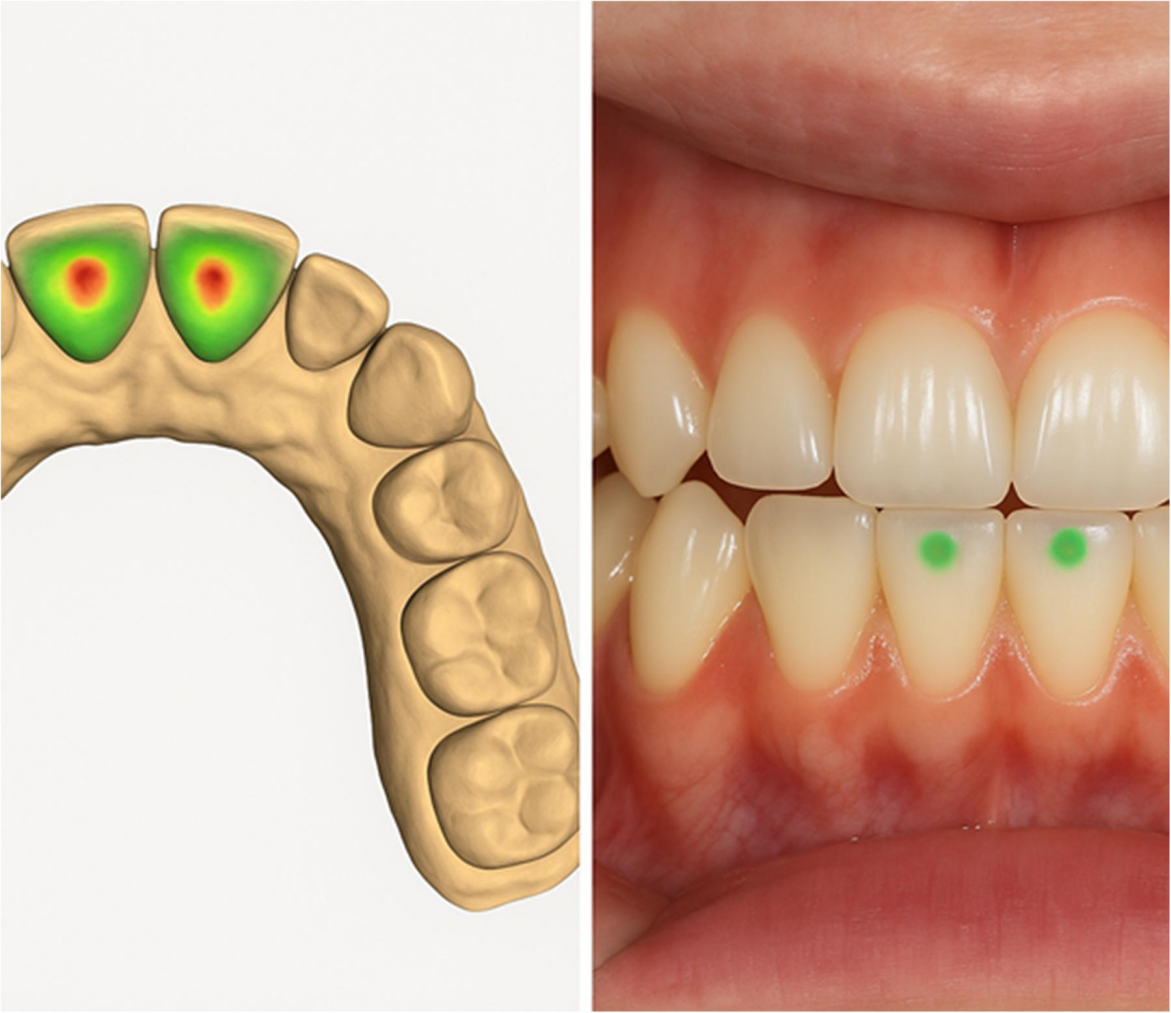
The conceptual occlusal verification stage within the proposed DUCU framework, showing the theoretical correspondence between the functional wax-up and antagonist contacts. These visualizations are AI-generated for explanatory purposes and do not represent clinical data.

**Figure 8:**
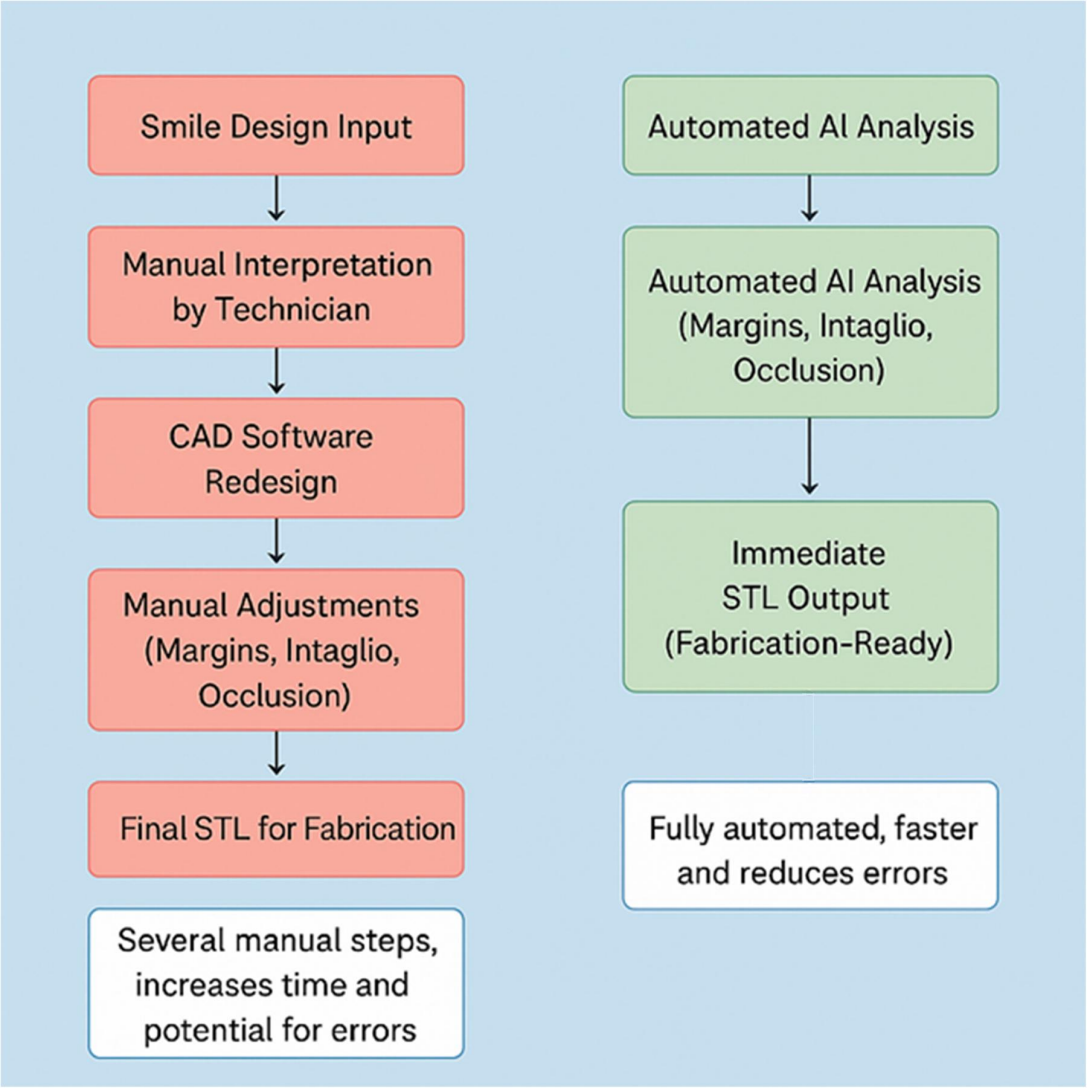
Comparative workflow illustration.

Following the simulated workflow illustrated in Figure 7, the next theoretical stage involves the verification of occlusal correspondence between the AI-generated restoration and the antagonist dentition. This conceptual validation step is represented in Figure 8, emphasizing how DUCU could integrate functional assessment into its digital workflow once implemented.

These simulated illustrations are intended to demonstrate the envisioned clinical translation of DUCU's theoretical framework, supporting its hypothesis of integrating aesthetics and functionality within a unified digital process.

## 3 Discussion

The introduction of digital smile design has revolutionized aesthetic dentistry by allowing motivational treatment simulations. However, these have remained largely estranged from the environment of CAD/CAM restoratives. At present, aesthetic mock-ups using software, for example, SmileCloud or exocad's TruSmile Video (21), are void of functional anatomy, which must be created separately within the CAD software, leading to added laboratory costs and inconsistency. DUCU bridges this gap by automatically transitioning between simulation and restoration (Figure 8).

SmileCloud, despite its excellent visual communication and real-human tooth library, does not have automatic CAD-ready restorative files. It needs a technician interpretation and CAD overlay to function. exocad's TruSmile Video (21), which was launched at the International Dental Show (IDS) 2025, too, provides dynamic smile animations eliciting emotional engagement; however, it does not create STL files or automatically generate a wax-up. It's a strong device for case acceptance—not for the creation of design.

3Shape's Smile Design and Dental System (22) are the ones that best reach the aesthetic-functional gap. They have a seamless 2D planning and 3D CAD fabrication workflow. Although margin detection, intaglio surface model generation, and final wax-up fabrication still require expert technician input, milling and pressing are becoming increasingly automated. It is a system that is comprehensive yet not fully automatic.

Instead, full AI-based automation of margin identification, intaglio generation, and occlusion-aware design (13) is proposed, leading to STL files that are print-ready with no technician rework. This could save laboratory time, increase digital accuracy, and expand the availability of high-end smile design.

To align the DUCU framework with clinical performance expectations, a structured set of evaluation and validation metrics has been conceptually defined. The primary indicators focus on the accuracy and functional fidelity of AI-generated restorations, including margin detection accuracy expressed as the mean deviation in millimeters compared with expert manual references, intaglio surface fit precision assessed through three-dimensional deviation mapping, and the occlusal correspondence index reflecting the percentage match between predicted and reference contact points within a virtual articulator environment.

The secondary indicators address workflow efficiency and user perception, considering the reduction in laboratory time per case, the decrease in manual technician interventions, and the clinician satisfaction score measured through structured feedback. Collectively, these parameters will serve as benchmarks for future validation studies, guiding the empirical assessment of DUCU's clinical applicability once the prototype becomes operational.

The regulatory implications are also relevant in AI-based clinical solutions such as DUCU. AI validation should be based on extensive testing to prove clinical robustness, consistency, and safety. Regulatory agencies including the FDA (USA) and CE mark (European Union) will evaluate AI-derived outputs, requiring robust evidence from controlled and real-world studies. We need to maintain these systems with auditable databases, continuous documentation of training and testing, and standard operating procedures for risk and mistake mitigation. Adherence to HIPAA and GDPR, as well as other local data protection laws, is also mandated when processing sensitive patient data, which requires ensuring the impenetrability of cybersecurity and infrastructure security.

Placed in the context of current limitations, which will help highlight the uniqueness of DUCU, the main blocks seen in the process of DSD-to-precise-print-wax include the lack of anatomic depth in DSD designs—the designs are veneers and not blocks of tissue, aesthetics element rather than volume basics (functionalism–inclusivism). Furthermore, software technologies are fragmented, meaning that the SmileCloud or DSD App networks are not integrated with CAD/CAM systems such as exocad (2) or 3Shape (22) at a detailed, editable level. This is further complicated by interdisciplinary complexity as successful wax-ups depend on deep integration of parameters such as tooth preparation margins, periodontal health, functional occlusion, and bone volume which have not been addressed in practically feasible manners in the current DSD systems.

On the other hand, DUCU is the first to alleviate such constraints through end-to-end automation—we achieve full automation in comparison to even full-lab solutions such as 3Shape (22), which only automate margin-based or intaglio generation or occlusion-aware design and require technicians to rework the designs. DUCU democratizes advanced CAD/CAM functions, enabling a general dentist to fabricate high-quality mock-ups or preparations without high-level CAD knowledge. Furthermore, DUCU's scalability can help to develop a unified AI-driven platform where patients, dentists, and laboratories collaborate simultaneously on the same case models. In contrast to 3Shape with separate tools tied together by workflows, DUCU provides intelligent design end-to-end, combining full-care design, margin marking, preparation design, and final STL output file in a unified AI-centered loop with no need for human rethinking, thus increasing workflow efficiency and restorative accuracy (23).

## 4 Conclusions

The distinction of smiling in the emotional smile simulations from the functional restorative workflow is a barrier to the realization of digital prosthodontics. DUCU represents a system of unified dental care (UDC)—a system in which aesthetic simulations can be efficiently converted to functional, printable, and even manufacturable restorations. Combining AI computer vision with CAD/CAM and collaborating in the cloud, DUCU offers the industry the first-of-its-kind platform that integrates emotional planning with clinical execution.

Although still theoretical, DUCU represents a strong foundation for overcoming the issue of manual duplication, improving accuracy, and providing broader access to high-end smile designs. Delivering on this vision will require collaboration across disciplines, sound clinical research, and continued technical innovation. However, its potential represents a significant step in the development of digital dentistry.

## Data Availability

The original contributions presented in the study are included in the article/Supplementary Material; further inquiries can be directed to the corresponding author.
